# Wide-field fluorescence navigation system for efficient miniature multiphoton imaging in freely behaving animals

**DOI:** 10.1117/1.NPh.12.2.025018

**Published:** 2025-06-27

**Authors:** Runlong Wu, Yukun Sun, Zeyu Hao, Chunzhu Zhao, Lishuang Feng, Aimin Wang, Heping Cheng

**Affiliations:** a Beijing Information Science and Technology University, School of Instrumentation Science and Opto-electronics Engineering, Beijing, China; b Beijing Municipal Education Commission, Beijing Laboratory of Biomedical Imaging, Beijing, China; c Beihang University, School of Instrumentation and Optoelectronic Engineering, Beijing, China; d Peking University, Institute of Molecular Medicine, College of Future Technology, Peking-Tsinghua Center for Life Sciences, National Biomedical Imaging Center, State Key Laboratory of Membrane Biology, Beijing, China; e Peking University, School of Electronics, Beijing, China; f Peking University, State Key Laboratory of Advanced Optical Communication System and Networks, Beijing, China

**Keywords:** miniature two-photon microscope, miniature three-photon microscope, freely behaving animals, wide-field fluorescence microscope, neuronal imaging, neurophotonics

## Abstract

**Significance:**

Miniature multiphoton microscopy has revolutionized neuronal imaging in freely behaving animals. However, its shallow depth of field—a result of high axial resolution—combined with a limited field of view (FOV), makes it challenging for researchers to identify regions of interest in three-dimensional space across multimillimeter cranial windows, thereby reducing the system’s ease of use.

**Aim:**

We aimed to develop a multimodal imaging platform with enhanced guidance and a standardized workflow tailored for efficient imaging of freely behaving animals.

**Approach:**

We present a wide-field fluorescence navigation system (WF-Nav) featuring a 90-mm working distance, a 4-mm FOV, and single-cell resolution, enabling rapid and precise localization of designated regions. By seamlessly integrating this navigation system with our prior miniature multiphoton microscopes, we established a multimodal platform that supports versatile imaging modalities and seamless transitions to two- or three-photon imaging. Building on this integration, we developed a streamlined workflow for efficient, user-friendly imaging in freely behaving mice.

**Results:**

We validated the system through large-FOV imaging (4 mm), dual-color imaging (920 and 1030 nm), and deep-brain neuronal imaging (up to 1 mm) in either awake mice or freely moving mice. The entire experimental procedure was completed in ∼20  min, achieving a 100% success rate (n=15).

**Conclusions:**

We have developed a comprehensive imaging platform that integrates a single-photon wide-field navigation system with miniature two-photon and three-photon microscopy, leveraging the strengths of each modality. Building on this platform, we established a streamlined workflow tailored for imaging freely behaving animals, markedly expanding its applicability and improving efficiency.

## 1 Introduction

Fluorescence imaging techniques, including single-photon (1P), two-photon (2P), and three-photon (3P) microscopy, have advanced significantly, driving progress in neuroscience research.^1^^–^^3^ In particular, miniature fluorescence microscopes (miniscopes) have been a game-changer in imaging neuronal structures and activity in freely behaving animals.^4^^–^^17^ Miniaturized single-photon microscopes (m1PMs), due to the high efficiency of 1P excitation, can use LED light sources to achieve illumination over several millimeters and a corresponding imaging field of view (FOV).^6^ However, due to the lack of optical sectioning capability, the *in vivo* resolution of m1PM is limited, making it challenging to visualize fine structures such as dendritic spines.^4^^–^^6^^,^^18^

Thanks to the intrinsic optical sectioning and deep-tissue penetration capabilities of 2P microscopy,^19^^–^^22^ miniaturized two-photon microscopes (m2PMs) have recently enabled imaging of neuronal synapses in the brains of freely moving animals.^8^^–^^15^ These impressive technological advancements have further expanded the imaging FOV to $1\times 0.8\;{\mathrm{mm}}^{2}$,^12^^,^^15^ enabled simultaneous three-color imaging, and achieved imaging depths of up to 854  μm.^15^ Furthermore, miniaturized three-photon microscopes (m3PMs) leverage the three-photon effect to achieve imaging depths exceeding 1 mm.^16^^,^^17^ However, the low-repetition-rate lasers used in three-photon microscopy limit the image pixel resolution during rapid, large-FOV imaging. Consequently, 1P, 2P, and 3P imaging modalities, each offering unique strengths in *in vivo* imaging, address different challenges and complement one another.

Despite their growing role in imaging freely behaving animals, miniaturized multiphoton microscopes still face several challenges in practice. The excellent optical sectioning of multiphoton microscopy results in a shallow depth of field (3 to 20  μm, depending on axial resolution), and its inherently low 2P excitation efficiency restricts the imaging FOV ($<1\;{\mathrm{mm}}^{2}$ without stitching).^8^^–^^15^ Without guidance, locating the designated region of interest (ROI) in three-dimensional space across multimillimeter cranial windows or graded index (GRIN) lens windows remains challenging. For inexperienced researchers, identifying the ROI and mounting the miniaturized microscope onto the mouse’s head can take tens of minutes or even several hours, significantly limiting their further adoption in freely behaving animals.

**Fig. 1:**
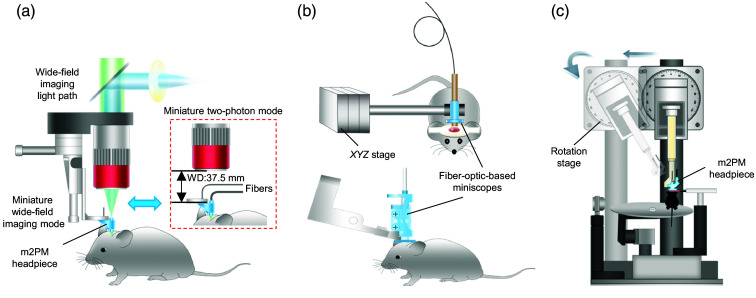
Representative strategies for mounting miniature microscopes on mice. (a–c) Mounting methods for miniature microscopes on mice used in previous studies: (a) Ref. 8, (b) Ref. 10 (top) and^7^ (bottom), and (c) Ref. 11. In panel (a), fibers represent the excitation and emission fibers. WD, working distance; Miniscopes, miniature fluorescence microscopes; m2PM, miniature two-photon microscope.

To address this issue, Zong et al^8^ combined a wide-field (WF) objective with the miniature objective of the m2PM headpiece (FHIRM-TPM), creating a WF fluorescence imaging configuration for ROI localization [Fig. 1(a)].^8^ Nevertheless, due to the limited FOV (∼150  μm) of the miniature objective, it is still difficult to identify ROIs within a multimillimeter cranial window. In addition, when switching to m2PM imaging, careful installation of the collection optical fiber is required. Due to the limited working distance (WD: 37.5 mm) of the WF objective, a larger bend in the optical fiber is necessary, which makes the operation more challenging [Fig. 1(a)]. Li et al^10^ and Dussaux et al^7^ used a three-axis translation stage to stabilize fiber-optic–based miniscopes for sample localization [Fig. 1(b)]. More recently, Zong et al^11^ incorporated a rotation stage to hold the m2PM headpiece, enabling angle adjustments for better alignment with specific brain regions [Fig. 1(c)]. Despite these advancements, rapidly locating ROIs in three-dimensional brain tissue remains a challenge, imposing a steep learning curve for new researchers. In addition, the WD of recent m2/3PMs is less than 2 mm, making them highly susceptible to specimen or objective lens damage when a z-axis stage is used to locate the focal plane, especially for inexperienced users. Overall, there is an urgent need to develop a multimodal imaging platform with enhanced guidance and a standardized workflow tailored for efficient imaging of freely behaving animals.

Here, we develop a 1P WF fluorescence navigation system (WF-Nav) and integrate it with miniature 2P and 3P microscopes, forming a multimodal fluorescence imaging platform. Building on this, we establish a streamlined workflow for brain imaging in freely behaving animals. The WF-Nav consists of a multicolor WF microscope and a multifunctional adapter, featuring a 9-cm WD, a 4-mm imaging FOV and single-cell resolution. The adapter, designed to prevent collision damage, enables seamless switching between WF microscopy and m2PM or m3PM, facilitating multimodal imaging and precise navigation of the designated ROIs. The system was validated through large-FOV 1P imaging in awake mice, as well as 2P dual-color and 3P deep-brain neuronal imaging in freely moving mice. The entire process was completed in ∼20  min, significantly enhancing imaging efficiency and experimental success rates.

## 2 Materials and Methods

### 2.1 m2PM System of the Multimodal Fluorescence Imaging Platform

The platform was equipped with two femtosecond lasers with wavelengths of 920 nm (Seed-FL-920-S1-80, Fatonics Technology, China) and 1030 nm (Seed-FL-1030-S1-80, Fatonics Technology, China). These lasers had a maximum average power of 1W, a pulse width of ∼120  fs, and a repetition rate of 80 MHz. An external half-wave plate (SAQWP05M-700, Thorlabs, USA) and an Acousto-Optic Modulator (AOM) (MT110-B50A1.5-IR-Hk, AA Sa., Orsay Cedex, France) were placed in the respective optical paths to rapidly control the laser power. Tunable beam expanders (TVS-BEP-0.5/2×-B, Transcend Vivoscope, China) were used to adjust the beam diameter for efficient fiber coupling. The two laser beams were combined using a mirror and a dichroic mirror (DMSP950, Thorlabs, USA), then coupled into an anti-resonant hollow-core fiber (AR-HCF) via a doublet (354171-B, LightPath Technologies, USA), and transmitted to the m2PM headpiece. The headpiece, weighing 2.6 g, was based on our prior study and had a lateral resolution of 0.79  μm, an axial resolution of 7.04  μm, and a FOV of $500\times 425\;\mu m^{2}$.^15^ By adjusting the 920-nm and 1030-nm laser dispersion, the positive dispersion induced by optical elements was compensated, achieving an exit dispersion close to 120 fs under the objective. The excited fluorescence was collected into a supple fiber bundle (SFB) by the miniature objective and an aspherical condenser and then transmitted by the SFB to a photomultiplier tube (PMT) module. The effective optical diameter of the SFB was 1 mm, with an NA of 0.57. In the PMT module, the fluorescence was first collimated by a collimating lens and passed through a low-pass filter (SP-700-25, Transcend Vivoscope, China) to remove the stray laser light. Subsequently, green and red channels of fluorescence were separated by a dichroic mirror set (DM-SP556-25×36, Transcend Vivoscope, China) and directed into two PMTs (H10770PA-40, HAMAMATSU, Japan) through a band-pass filter set (BP-520/70-25 and BP-625/90-25, Transcend Vivoscope, China), respectively.

### 2.2 m3PM System of the Multimodal Fluorescence Imaging Platform

A noncollinear optical parametric amplifier (NOPA, Spectra-Physics, USA), pumped by a one-box regenerative amplifier (Spirit, Spectra-Physics, USA), generated femtosecond laser pulses centered at 1300 nm, with a maximum average power of ∼500  mW and a repetition rate of 408 kHz. To control the optical power, we used a half-wave plate (HWP, WPA2415-900-2100, Union Optic, China) and a polarizing beam splitter (PBS124, Thorlabs). The laser was coupled into a hollow-core fiber (HC-1300) using a specific lens (AC127-019-C, Thorlabs, USA) and transmitted to the m3PM headpiece. The headpiece, weighing 2.2 g, was based on our prior study and had a lateral resolution of 0.97  μm, an axial resolution of 7.2  μm, and a FOV of $400\times 400\;\mu m^{2}$.^16^ The scattered fluorescence was collected by the SIMO objective and an Abbe condenser and then transmitted through an SFB to the PMT module. The SFB had an effective optical diameter of 1.7 mm and an NA of 0.57. Within the PMT module, fluorescence was first collimated by a collimating lens and passed through a low-pass filter (FF01-790/SP-25, Semrock, USA) to block the laser light. A dichroic mirror (TVS-FP-475-750-1, Transcend Vivoscope, China) then separated the green fluorescence and third-harmonic generation (THG) signals, directing them to their respective PMTs (H10770PA-40, HAMAMATSU, Japan) through a bandpass filter (FF01-520/70-25 and FF01-442/42-25, Semrock, USA), respectively.

### 2.3 Animals

All the animal housing and experimental procedures were approved by the Peking University Animal Use and Care Committee and complied with the Association for Assessment and Accreditation of Laboratory Animal Care standards. Male C57BL/6 wild-type mice (8 to 16 postnatal weeks) were used in these experiments. Animals were housed with a 12-h light-dark cycle at 22°C with 50% humidity and had free access to food and water.

### 2.4 Virus Injection

Mice were anesthetized with 1.5% isoflurane in air at a flow rate of 0.4 L/min and mounted on a stereotactic frame. AAV2/9-hSyn-GCaMP6f-WPRE-hGH-pA was injected into the postsubiculum (AP, −4.2  mm; ML, −2  mm; DV, −0.4  mm; −0.7  mm; −1.2  mm), and AAV2/9-hSyn-GCaMP6s-WPRE-hGH-pA, rAAV-GfaABCID-mCherry-WPRE-SV40pA, and rAAV-hSyn-mCherry were injected into the M1 (AP, 0.5 mm; ML, 1.2 mm; DV, −0.3  mm). AAV viruses were slowly injected into the targeted brain regions at a rate of 10  nL/min. The injection needle was left in place for an additional 5 min after the injection.

### 2.5 Surgical Procedure for Cranial Window Preparation

One week after the virus injection, a 4.3-mm-diameter craniotomy was made. Mice were anesthetized with isoflurane, and a 4.3-mm piece of the skull was carefully removed with the dura intact over the targeted cortex (M1: AP, 0.5 mm; ML, 1.2 mm; postsubiculum: AP, −4.2  mm; ML, −2  mm). A piece of glass coverslip (4.3-mm diameter, TJ-001, 85 to 115μm, Optowide Technologies Inc.) was placed on the craniotomy and sealed with cyanoacrylate and dental cement.

### 2.6 Data Processing and Analysis

We used the open-source software ImageJ for averaging, cropping, registration, pseudocolor-coding, and three-dimensional projection of raw images. Three-dimensional reconstruction figures and movies were made using Imaris (Bitplane). ${\mathrm{Ca}}^{2+}$ imaging data were analyzed by Suite2p.^23^ We used Image Stabilizer (ImageJ) to correct motion artifacts before processing timeseries images. We then used an annular ring subtraction method^24^ to present ${\mathrm{Ca}}^{2+}$ signals as relative fluorescence changes (ΔF/F).

## 3 Results

### 3.1 System Design of the Multimodal Fluorescence Imaging Platform

**Fig. 2:**
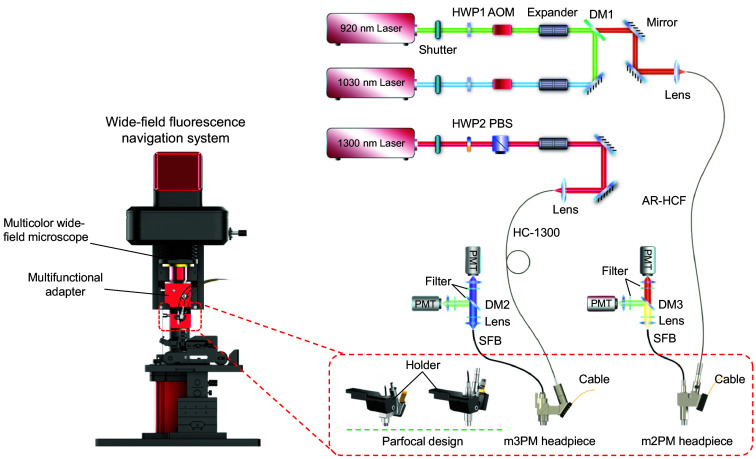
System design of the multimodal fluorescence imaging platform. Overall schematic of the multimodal fluorescence imaging platform. HWP, half-wave plate; AOM, acousto-optic modulator; DM, dichroic mirror; AR-HCF, anti-resonant hollow-core fiber for 760 to 1060 nm waveband transmission; PBS, polarizing beam splitter; HC-1300, hollow-core optical fiber for 1300 nm transmission; SFB, supple fiber bundle; PMT, photomultiplier.

The overall configuration of the multimodal fluorescence imaging platform was consistent with that reported in our previously developed microscopes,^15^^,^^16^ incorporating important improvements and modifications. In our setup, we integrated the WF-Nav with m2PM and m3PM (Fig. 2). For m2PM, 920- and 1030-nm femtosecond lasers (repetition rate: 80 MHz) coupled into a single antiresonance hollow-core fiber (AR-HCF, bandwidth: 700 to 1060 nm)^15^ and delivered to the headpiece. For m3PM, a 1300-nm femtosecond laser (repetition rate: 408 kHz) was coupled into a hollow-core fiber (HC-1300).^16^ Both 2P and 3P lasers feature dispersion compensation capabilities. Building on our prior work of FHIRM-TPM 3.0^15^ and m3PM,^16^ we redesigned the headpieces by standardizing the mechanical dimensions to share the same headpiece holder and mounting mechanism, ensuring a parfocal design and a consistent operational workflow. Fluorescence signals were transmitted via a supple fiber bundle (SFB) to two photomultiplier tubes (PMTs), separated by a dichroic mirror (DM) for dual-channel detection (Fig. 2).

To fully leverage the advantages of 1P excitation, the WF-Nav features a large FOV and single-cell resolution, enabling rapid identification of designated ROIs. Once the target area is located, the system seamlessly switches to m2PM or m3PM modality for high signal-to-background ratio and deep neuronal imaging. Finally, the m2PM or m3PM headpiece was cemented to the mouse’s head, allowing it to be released from the head-fixed treadmill for free movement.

### 3.2 Design of the WF-Nav

**Fig. 3:**
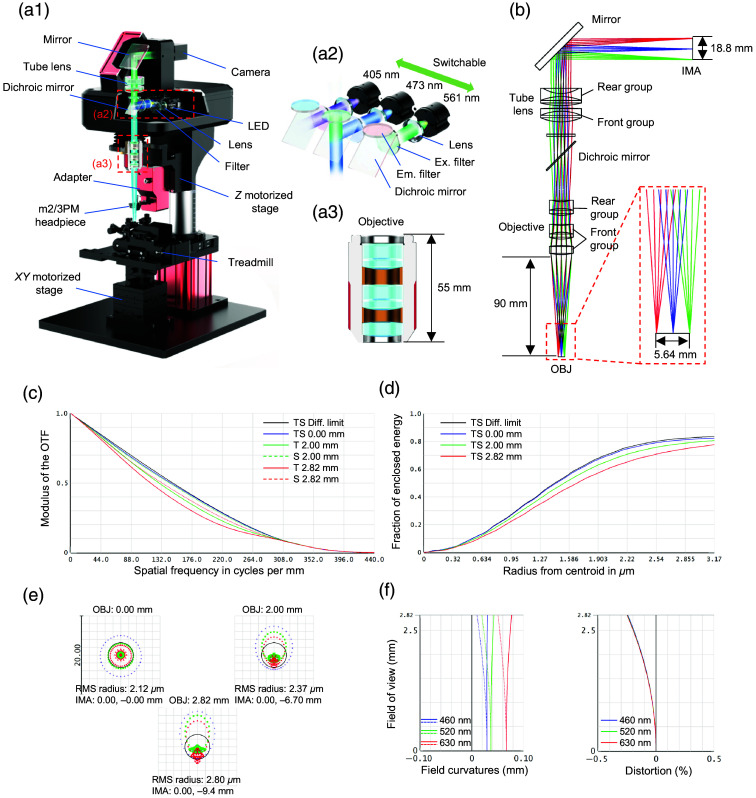
Design of the WF-Nav. (a1) Schematic of the WF-Nav. (a2) Schematic of the excitation light path. Three separate filter sets were used for the 405 nm (Ex.: 405±10  nm; Em.: 460±25  nm; DM edge: 525 nm), 473 nm (Ex.: 482±9  nm; Em.: 520±14  nm; DM edge: 495 nm), and 561 nm (Ex.: 560±40  nm; Em.: 630±38  nm; DM edge: 585 nm) light paths. The handle was used to manually switch between excitation paths. (a3) Cross-section view of the objective. (b) Optical layout of the WF fluorescence microscope. The working distance of the objective was 90 mm. The OBJ FOV was Φ5.64 mm, and the IMA FOV was Φ18.8  mm. (c) Variation of Modulus of the Optical Transfer Function (OTF) with spatial frequency at field points of 0, 2, and 2.82 mm. (d) Fraction of enclosed energy of the focal spot at field points of 0 mm, 2 mm, and 2.82 mm. (e) Spot diagram showing the root mean square (RMS) radius, which was 2.12  μm at the center and 2.80  μm at (0, ±2.82  mm). (f) Field curvatures and distortion percentages across different FOVs at 460, 520, and 630 nm. LED, light-emitting diode; Ex., excitation; Em., emission; Diff. limit, diffraction limit; TS, tangential and sagittal; T, tangential; S, sagittal; OBJ, object plane; IMA, image plane; FOV, field of view.

The WF-Nav consists of a multicolor large-FOV microscope and a modality-switching adapter [Fig. 3(a1)]. It employed three LEDs (405 nm; 1.3 W; divergence angle [DA], 120 deg; 473 nm; 1 W; DA, 80 deg; 561 nm; 0.95 W; DA, 120 deg) as excitation sources, with a high number-capture (NA) aspheric condenser lens (focal length, 15 mm; NA, 0.534) to collimate the LED light [Fig. 3(a2)]. Each excitation path included a corresponding filter set. To enable large FOV navigation and provide ample space for seamless switching to multiphoton headpieces, we designed a long-WD and low-magnification dry objective [Figs. 3(a3) and 3(b)]. This objective (3.3×; NA, 0.1) features a reverse telephoto design, with a front group of two cemented doublet lenses providing negative optical power and a rear group consisting of a single spherical lens. This configuration shifts the principal plane rearward to correct chromatic aberration and allows for a WD longer than the effective focal length while maintaining the NA. As a result, it delivers a 90-mm WD and a 60-mm focal length. To our knowledge, this objective offers the longest WD for its magnification and NA at this scale. The tube lens was designed with a telephoto configuration. It consists of a front positive and rear negative cemented doublet group, enabling a long focal length (200 mm) while shortening the distance to the camera (147 mm). These optical designs made the system more compact. Both the objective and tube lenses are optimized for spherical and chromatic aberration correction (400 to 700 nm).

The system achieved a maximum imaging FOV of Φ5.64  mm. As shown in Fig. 3(c), the spatial frequency corresponding to a modulation transfer function (MTF) value of 0.15 reached 270-line pairs per millimeter (equivalent to a 3.7-μm feature size) in the central field. In addition, the 80% energy concentration of the spot diagram and root mean square (RMS) radius remained within the Airy disk radius [Figs. 3(d) and 3(e)]. These simulation results indicate that the optical design is close to the theoretical diffraction limit. Across the full FOV, the tangential and sagittal field curvatures were both optimized to within 0.017 mm, and the distortion was controlled below 0.25% [Fig. 3(f)].

**Fig. 4:**
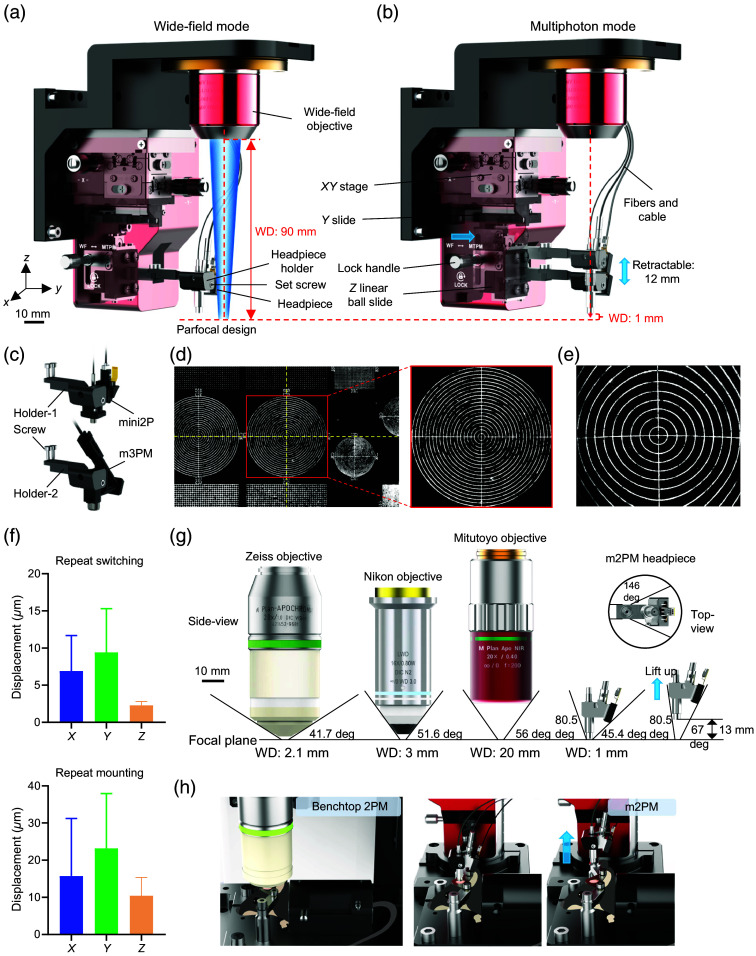
Design and testing of the multifunctional adapter and comparison of approach angles across different objectives. (a) Schematic of the adapter switched to the WF imaging mode. WD: working distance. (b) Schematic of the adapter switched to the miniature multiphoton imaging mode. The Z linear ball slide was used to prevent collision damage and increase the approach angle. The lock handle used threading to secure the Z slide, allowing it to remain at any desired height. (c) Redesigned interchangeable headpiece holders (holders 1 and 2) to mount the mini2P developed by Zong et al.^11^ and the m3PM developed by Klioutchnikov et al.,^17^ enabling alignment of their optical axes with the WF objective. (d) Switching between WF (left) and m2PM (right) modes to image the same FOV and focal plane on the fluorescent target. The center panel shows a magnified view of the red-boxed region on the left. Yellow dashed crosshairs indicate the imaging center. (f) Top: absolute errors in repeated mode switching (WF → m2P → WF → m2P), measured after calibration. X and Y represent m2PM FOV displacement; Z represents m2PM focal plane shift. Mean ± SD: 6.9±4.8  μm (X), 9.4±5.9  μm (Y), 2.3±0.51  μm (Z); n=20. Bottom: absolute errors in repeated remounting of the m2PM headpiece relative to its original position. Mean ± SD: 15.8±15.5  μm (X), 23.2±14.8  μm (Y), 10.4±4.9  μm (Z); n=20. (g) Top: top-view of the approach angle of the m2PM headpiece. Bottom: side-view of the approach angle of representative benchtop two-photon objectives (Zeiss 421452-9681-000, Nikon N16XLWD-PF and Mitutotyo M Plan APO NIR 20x) compared with the m2PM headpiece. The approach angles were measured at a 3.6-mm FOV at the focal plane. Bottom right: approach angle of the headpiece when raised by 12 mm. (h) Left and middle: schematic diagram of the head-fixed imaging experimental setup with a Zeiss objective (left) and the m2PM headpiece (middle). Right: schematic diagram of the m2PM headpiece raised via the Z slide and secured by the lock handle.

To enable seamless switching between imaging modalities, we designed a multifunctional adapter [Fig. 4(a)]. The adapter, connected to the WF objective, is mounted on a Z-axis motorized stage for focal adjustment. The standardized m2/3PM headpiece design allows for secure attachment to the same holder, ensuring parfocal imaging when switching between multiphoton and WF modalities [Figs. 4(a) and 4(b)]. The 90-mm WD objective provided ample space for maneuvering the headpiece, optical fibers, or adding a detachable electric-tunable lens module.^9^ The headpiece holder is mounted on a lightweight linear ball slide with minimal sliding resistance (∼0.1  N) and a 12-mm retractable distance, protecting the front lens and specimen from collision damage [Fig. 4(b)]. By replacing the headpiece holder, the system can accommodate different headpieces, such as the mini2P^11^ and miniature 3PM^17^ [Fig. 4(c)].

For calibration, a fluorescent target (TVS-EasyFOV-02, Transcend Vivoscope, China) was first imaged in WF mode, then switched to m2/3PM mode while adjusting the XY stage to ensure coaxial optical alignment. The distance between the camera and the tube lens was finely adjusted to align their focal planes. These adjustments allowed both modalities to share the same central FOV and focal plane [Figs. 4(d) and 4(e)]. We further quantified the repeatability of mode switching and headpiece remounting [Fig. 4(f)]. The maximum absolute errors in XY and Z were less than 60 and 20  μm, respectively, demonstrating reliable FOV targeting. These errors can be further minimized by reapplying the calibration procedure. In addition, thanks to the slim size of the miniature headpiece and the retractable design of the holder, it provided a broader approach angle compared with benchtop objectives in most directions [Fig. 4(g)], resulting in a larger specimen observation range [Fig. 4(h)]. This design dramatically improves coarse localization of the specimen. The list of WF-Nav components is provided in the Supplementary Material (Table S1).

### 3.3 Performance Testing of the WF Fluorescence Microscope

**Fig. 5:**
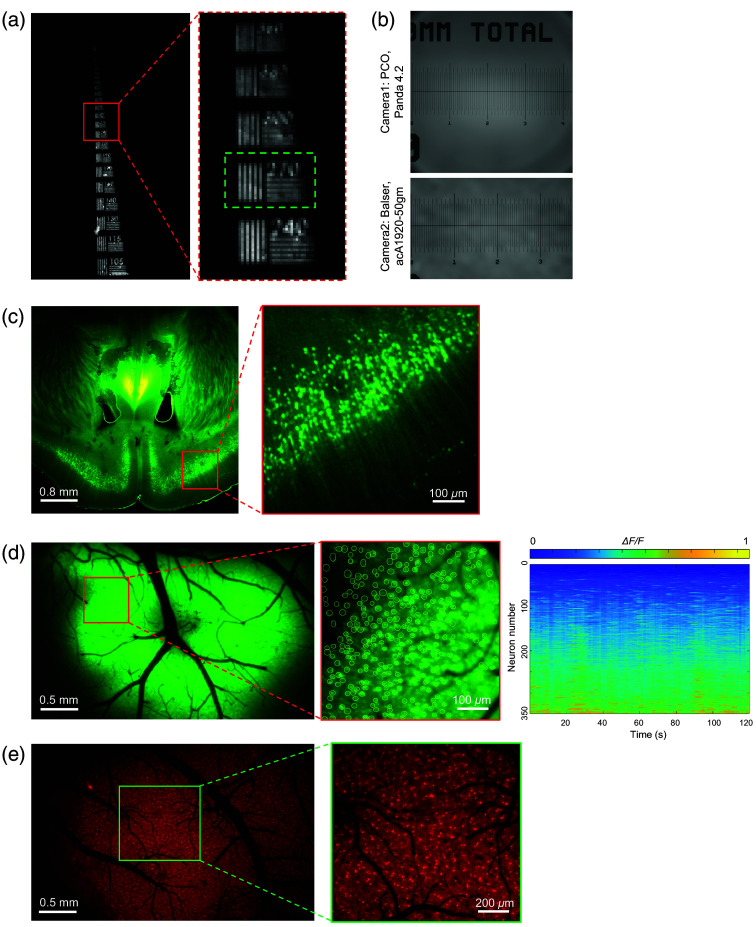
Performance testing of the WF fluorescence microscope. (a) Spatial resolution test using a USAF1951 resolution test target (#55-622, Edmund Optics) illuminated by a 520-nm LED. The expanded view is shown on the right. The green-dashed box highlights 270 line pairs. (b) Imaging FOV measurements using Panda 4.2 (FOV, $4.28\times 4.28\;mm^{2}$) and acA1920-50gm (FOV: $3.76\times 2.35\;mm^{2}$) cameras, with a scale test target (R1L3S5P, Thorlabs) for calibration. (c) Imaging of a Thy1-YFPH brain slice. The expanded view is shown on the right. (d) Left: average intensity projections from M1 neurons expressing GCaMP6s. Right: raster plots of representative neuronal $Ca^{2+}$ activity from neurons in the red-box region of the left panel, with an expanded view shown in the middle. (e) Left: average intensity projections from M1 astrocytes expressing mCherry. The expanded view is shown on the right. Data in panel (c) were recorded with the Panda 4.2 camera, whereas images in panels (d) and (e) were captured using the acA1920-50gm. Data were obtained from representatives of three similar experiments.

Next, we assessed the optical performance of the WF fluorescence microscope. Resolution testing with a USAF 1951 target (#55-622, Edmund Optics, USA) achieved 270-line pairs per millimeter, in line with our design [Fig. 5(a)]. Imaging of a scale target (R1L3S5P, Thorlabs, USA) yielded FOVs of $4.28\times 4.28\;{\mathrm{mm}}^{2}$ and $3.76\times 2.35\;{\mathrm{mm}}^{2}$ with different cameras [Fig. 5(b)].

We imaged Thy1-YFPH brain slices and clearly identified neurons and axons [Fig. 5(c)]. *In vivo* $Ca^{2+}$ imaging was performed at 470 nm, and morphology imaging at 561 nm, capturing a large cohort of 3804 GCaMP6f-labeled neurons [Fig. 5(d), Video 1, MP4, 11.5 MB] and 4650 astrocytes in mCherry-expressing mice [Fig. 5(e)]. These results demonstrated the WF microscope’s capability for multicolor, single-cell resolution, and high-throughput imaging, making it highly effective for rapid target ROI navigation.

### 3.4 m2PM Imaging Workflow in Freely Behaving Mice

**Fig. 6:**
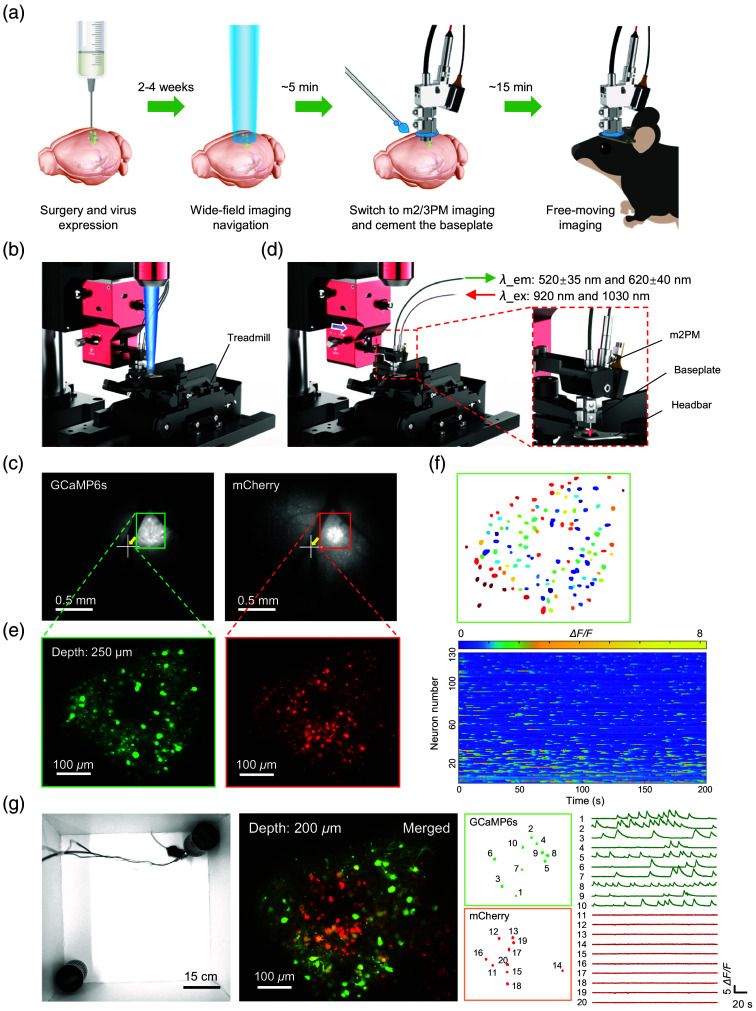
m2PM imaging workflow in freely behaving mice. (a) Entire workflow of m2PM or m3PM imaging in freely behaving mice. (b) Schematic of the WF fluorescence imaging mode. (c) WF fluorescence microscopy imaging of a head-fixed mouse in the M1 region expressing GCaMP6s and mCherry. The green and red boxes denote the regions designated for further two-photon imaging. The white cross indicates the center of the FOV. (d) Schematic of the m2PM imaging mode. $\lambda_{\mathrm{em}}$, emission wavelength; λ_ex, excitation wavelength. (e) Two-photon imaging of GCaMP6s (green) and mCherry (red) in the target regions from panel (c). (f) Top: segmentation maps of 130 identified neurons from the left side of panel (e). Bottom: raster plots of representative neuronal $Ca^{2+}$ activity from the segmented neurons. (g) Left: representative snapshot of a mouse in a 60-cm social behavior box. Middle left: average intensity projections of merged M1 neurons expressing GCaMP6s and mCherry. Middle right: segmentation maps of twenty representative identified neurons. Right: representative time courses of GCaMP6s Ca2+ activity (green) and mCherry fluorescence (red) in the segmented areas (marked with numerals in corresponding images). Data were obtained from five animals and are representative of fifteen similar experiments.

We established a standardized imaging workflow to improve the efficiency of m2PM imaging in freely moving animals [Fig. 6(a)]. First, virus injection and surgical procedures, such as cranial window implantation or GRIN lens insertion, are performed, followed by a 2- to 4-week period for full viral expression and recovery. In one instance, we labeled GCaMP6s and mCherry in neurons of the primary motor cortex (M1), and the mice were then secured on a treadmill beneath the WF-Nav [Fig. 6(b)]. Next, the designated ROI was identified and aligned to the center of the FOV using the WF microscope [Fig. 6(c)]. The m2PM modality was then switched via the adapter for dual-color two-photon imaging at 920 and 1030 nm [Fig. 6(d)], followed by fine adjustments to the XY position and focal plane to confirm the final ROI within a few minutes [Fig. 6(e)]. In most cases, we also raised the target focal plane by ∼25  μm to compensate for the focal shift caused by cement shrinkage. Finally, the baseplate was cemented to the head-bar, which had been permanently installed on the animal’s head. After ∼10  min of curing, the mouse was released for free-moving imaging.

**Fig. 7:**
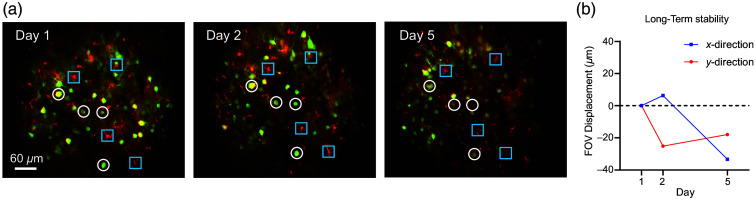
Recurring imaging of the same cohort of neurons and microglia over 5 days in a freely moving mouse. (a) Longitudinal imaging of neurons (green) and astrocytes (red) in M1 at 250-μm depth. White circles and blue squares mark the same cells across days. (b) FOV displacement over multiple days during the experiment shown in (a).

We validated the system by stably capturing neuronal ${\mathrm{Ca}}^{2+}$ signals and neuronal structures during single-box social behavioral exploration [Figs. 6(g) and 6(f), Video 2, MP4, 11.5 MB]. Typically, the headpiece was removed after the experiment, and following 2 to 3 days of habituation, it was reattached to the baseplate for the imaging experiment. The entire procedure took ∼20  min and boasted a 100% success rate (n=15), making it accessible even to inexperienced researchers, thereby improving the experimental efficiency and reducing the learning curve. In addition, we performed multiday recurring imaging of neurons and astrocytes in layer 2 of M1, demonstrating stable FOV registration with a shift of less than 33  μm across days without baseplate reinstallation [Figs. 7(a) and 7(b)].

### 3.5 m3PM Imaging in the Postsubiculum of Head-Fixed and Freely Exploring Mice

**Fig. 8:**
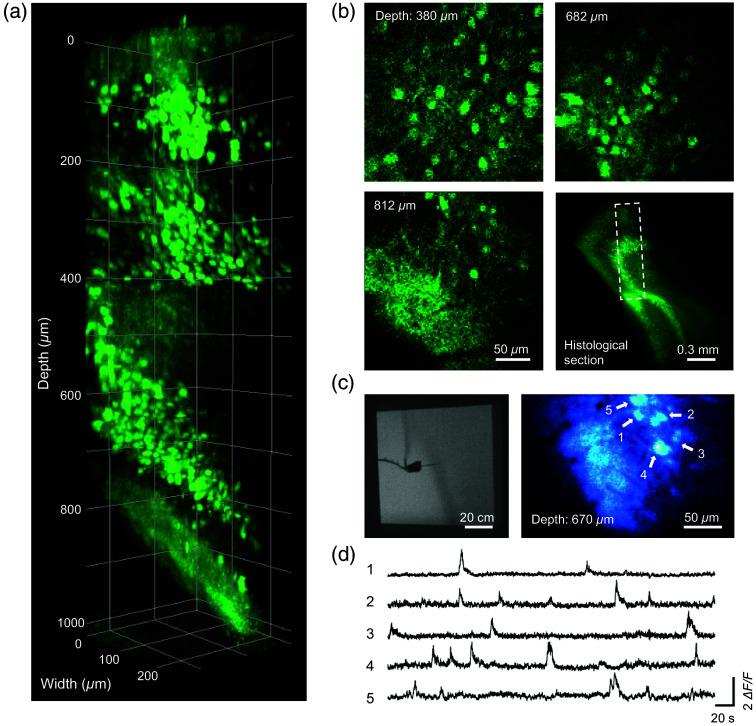
m3PM imaging in the postsubiculum of head-fixed and freely exploring mice. (a) Three-dimensional reconstruction of a 1020-μm stack of GCaMP6f-labeled neurons in the postsubiculum of a head-fixed mouse. (b) Left and top right: average projections of 50 x–y frames at representative depth. Bottom right: histological section of GCaMP6f-labeled neurons in the postsubiculum, with the dotted box indicating the stack imaging direction. (c) Left: representative snapshot of a mouse in an 80-cm square open-field box. Right: average m3PM images at a depth of 670  μm below the cortical surface. (d) Representative calcium traces from indexed neurons on the right side of panel (c).

Thanks to the standardized mechanical design, the m3PM headpiece can be mounted onto the holder. The adapter enables seamless switching from the WF mode to the three-photon imaging mode. Due to the limited imaging depth of WF microscopy, signals from deep layers are often undetectable. Although superficial blood vessels can offer some guidance, retaining a superficial virus injection site provides a more direct fluorescent landmark to aid in aligning with deeper regions along the same vertical axis. Neuronal activity in a head-fixed GCaMP6f-expressing mouse was recorded from the cortical surface to the postsubiculum at an imaging depth of 1020  μm [Figs. 8(a) and 8(b), Video 3, MP4, 4.75 MB]. The workflow for freely behaving imaging is similar to that of m2PM imaging. We conducted imaging in mice engaged in open-field behavior exploration and captured ${\mathrm{Ca}}^{2+}$ activity at a depth of 670  μm [Figs. 8(c) and 8(d), Video 4, MP4, 11.5 MB].

## 4 Discussion and Conclusion

In summary, we have developed a comprehensive imaging platform that integrates a 1P WF navigation system with miniature 2P and 3P microscopy, leveraging the strengths of each modality. Based on this platform, we established a streamlined workflow tailored for imaging freely behaving animals. The process began with large-FOV 1P WF imaging to facilitate precise navigation of target regions. Once the ROI was identified, the system was switched to the m2PM or m3PM modality. Finally, the headpiece was cemented to the animal, enabling data recording in a freely moving state. The entire procedure can be completed in ∼20  min and was therefore accessible to inexperienced researchers. The platform, encompassing WF-Nav, accommodates a diverse range of imaging paradigms, including various animal models (e.g., rats, marmosets, and songbirds), different tissues (e.g., the spinal cord, lungs, kidneys, and liver), diverse physiological states (e.g., head-fixed, anesthetized, and freely behaving), and multiple fluorescent indicators (e.g., DAPI, GFP, GCaMP, mCherry, and jRGECO1a). Moreover, the workflow extends to additional imaging modalities, such as m1PM and miniature ultrasound probes, offering a versatile, easy-to-use, and scalable platform for advancing *in vivo* imaging in freely behaving animals.

## 5 Appendix: Video Captions

The following videos are mentioned in the text:

**Video 1** Wide-field imaging of cortical neurons in head-fixed mouse (MP4, 11.5 MB [URL: https://doi.org/10.1117/1.NPh.12.2.025018.s1]).
**Video 2** Dual-color calcium imaging in freely exploring mouse (MP4, 11.5 MB [URL: https://doi.org/10.1117/1.NPh.12.2.025018.s2]).
**Video 3** 3D imaging from cortex to postsubiculum in head-fixed mouse (MP4, 4.75 MB [URL: https://doi.org/10.1117/1.NPh.12.2.025018.s3]).
**Video 4** Deep brain imaging in freely moving mouse using m3PM (MP4, 11.5 MB [URL: https://doi.org/10.1117/1.NPh.12.2.025018.s4]).

## Supplementary Material

10.1117/1.NPh.12.2.025018.s01

10.1117/1.NPh.12.2.025018.s1

10.1117/1.NPh.12.2.025018.s2

10.1117/1.NPh.12.2.025018.s3

10.1117/1.NPh.12.2.025018.s4

## Data Availability

Data underlying the results presented in this paper are not publicly available at this time but may be obtained from the authors upon reasonable request.
