# Supplementary material for: Wide-field fluorescence navigation system for efficient miniature multiphoton imaging in freely behaving animals
Source: Neurophotonics. 2025 Jun 27;12(2):025018. doi: 10.1117/1.NPh.12.2.025018 (PMC12204226; doi:10.1117/1.NPh.12.2.025018)
Supplement: Supplementary file 1 [file NPh_012_025018_SD001.pdf]

Supplemental Material

Wide-field fluorescence navigation system for efficient miniature multiphoton imaging in freely behaving animals

Runlong Wu, Yukun Sun, Zeyu Hao, Chunzhu Zhao, Lishuang Feng, Aimin Wang, Heping Cheng

Video 1 Wide-field imaging of cortical neurons in head-fixed mouse

Time-lapse imaging of GCaMP6s-labeled neurons in a head-fixed mouse using the wide-field microscope at a frame rate of 25 Hz. The imaging data were processed by subtracting the minimum projection of the stack and correcting for motion artifacts using the Image Stabilizer plugin (ImageJ). The FOV is 3.76×2.35 mm^2. The video is played at 4× speed.

Video 2 Dual-color calcium imaging in freely exploring mouse

Dual-color imaging of GCaMP6s-labeled neuronal Ca^{2+} activity (920 nm excitation) and mCherry-labeled neuronal morphology (1030 nm excitation) in the M1 during open-field exploring using the m2PM headpiece at a frame rate of 9.8 Hz (512 × 440 pixels). On the right, representative traces of 20 selected neurons are shown. The moving white line on the traces is synchronized with the behavior video and neuronal activities. The video is played at 3× speed.

Video 3 3D imaging from cortex to postsubiculum in head-fixed mouse

Three-dimensional imaging GCaMP6s-labeled neurons in a head-fixed mouse. A 1,020-μm stack was acquired from the cortical surface to postsubiculum, with 2-μm

intervals. The FOV is 250 × 250 μm^2. The imaging depth is displayed on the top left corner.

Video 4 Deep brain imaging in freely moving mouse using m3PM

m3PM achieved deep brain imaging in a freely behaving mouse. Left, video of a mouse freely moving in the 80-cm open-field box. Neurons expressing GCaMP6f at a depth of 670 μm were imaged at a frame rate of 8.35 Hz (256 × 256 pixels). The data were corrected for lateral motion artifacts and processed with a three-frame moving average with the Running Z Projector plug-in (ImageJ). Right, time courses of Ca^{2+} activity from indexed neurons expressing GCaMP6f in the x-y image. The optical power after the objective is 35 mW. The moving blue line on the traces was synchronized with the behavior video. The video is played at 10× speed.

Supplemental Tables

Table S1 List of components for WF-Nav system

Component	Vendor	Part#
**Camera**		
CMOS1	PCO Imaging (Shanghai)	panda 4.2
CMOS2	Basler	acA1920-50gm
**LED module**		
Violet LED	Shenzhen Silverlight Technologies	M3535N1UVS8U06-405
Blue LED	OSRAM (China) Lighting	GB CS8PM1.13
Green LED	OSRAM (China) Lighting	KP CSLNM1.F1
405/20nm bandpass filter	Transcend Vivoscope	3001644
482/18nm bandpass filter	Transcend Vivoscope	3001540
560/40nm bandpass filter	Transcend Vivoscope	3001641
Aspheric condenser lens	Thorlabs (Shanghai)	ACL1815U-A
LED PCBA control board	Transcend Vivoscope	Custom-fabricated by TVS
**Wide-filed microscope**		
Mirror	Transcend Vivoscope	MIR22037A

Objective lens	Transcend Vivoscope	Custom-fabricated by TVS
Tube lens	Transcend Vivoscope	Custom-fabricated by TVS
460/50nm bandpass filter	Transcend Vivoscope	3001646
425nm longpass dichroic mirror	Transcend Vivoscope	3001649
520/28nm bandpass filter	Transcend Vivoscope	3001659
495nm longpass dichroic mirror	Transcend Vivoscope	3001658
630/75nm bandpass filter	Transcend Vivoscope	3001643
585nm longpass dichroic mirror	Transcend Vivoscope	3001648
**Adapter**		
Linear rolling unit	THK	LS1027
Slider	Transcend Vivoscope	1001643
Stainless steel rail	HIWIN Technologies	MGWR5R33CM(E1=E2=6.5)
Rail-1	THK	VR1_50HX13Z
Rail-2	THK	VR2-120HX21Z
Flanged sleeve bearing	igus (Shanghai)	JFM-0608-08
Magnet D10×3 NdFeB Ni-plated	MISUMI Group	HXN10-3
Positioning pin	MISUMI Group	HPB-3X6-SUS
Compact displacement stages	SIGMAKOKI	TSDH-252WSR
**XYZ motor stage and controller**		
XYZ motor stage and controller	Transcend Vivoscope	1001404
**Other mechanical structures**		
Microscope body and other components	Transcend Vivoscope	Machined by TVS
